# The fruitfly *Drosophila melanogaster* as an *in vivo* model of ferroptosis

**DOI:** 10.1016/j.redox.2026.104418

**Published:** 2026-09-28

**Authors:** Li Zhang, Felix Schmidt, Fatih Yasar, Yasminn Akele, Bernhard Michalke, Konstantin Beckschäfer, Christoph Thiele, Eva-Maria Hanschmann, Vivek Venkataramani, Axel Methner

**Affiliations:** a University Medical Center of the Johannes Gutenberg-University Mainz, Institute for Molecular Medicine, Mainz, Germany; b Research Unit Analytical BioGeoChemistry, Helmholtz Center Munich-German Research Center for Environmental Health, Neuherberg, Germany; c Life and Medical Sciences (LIMES) Institute, University of Bonn, Bonn, Germany; d Lausitz Medical University - Carl Thiem, Biochemistry, Cottbus, Germany; e Department of Internal Medicine II, Medical Oncology, and Department of Neurosurgery, University Hospital Würzburg, Bavarian Cancer Research Center (BZKF), National Center for Tumor Diseases (NCT) WERA, Würzburg, Germany

**Keywords:** Ferroptosis, Drosophila, Iron homeostasis, *In vivo* model

## Abstract

Ferroptosis is an iron-dependent form of cell death involved in cancer, ischemia-reperfusion injury, inflammation, and neurodegeneration. It can be triggered in two ways: class 1 inhibitors such as erastin block system xc^−^, the cystine/glutamate antiporter composed of xCT and 4F2hc/CD98, causing glutathione depletion, whereas class 2 inhibitors such as RSL3 directly inhibit glutathione peroxidase 4, causing lipid peroxidation. Ferroptotic cell death is suppressed by radical-trapping antioxidants like ferrostatin-1. We investigated the effects of erastin and RSL3 on *Drosophila melanogaster* lifespan, iron metabolism, and lipid composition. Erastin and RSL3 dose- and diet-dependently shortened lifespan which was rescued by ferrostatin-1. Erastin increased the Fe^2+^/Fe^3+^ ratio and reduced ferritin-bound iron, and RSL3 remodeled lipid composition consistent with lipid peroxidation. Both effects occurred only in male *w*^*1118*^ flies although both sexes had similarly reduced lifespans. Total glutathione was unchanged by erastin; notably, the fly GPX4 ortholog Gtpx is a non-selenocysteine, thioredoxin-dependent peroxidase. Complete *Gtpx* knockout caused early lethality mirroring the embryonic lethality of constitutive GPX4 knockout in mice but was not affected by ferrostatin-1. Flies also lack a clearly defined xCT ortholog. Together, these results establish male *w*^*1118*^ flies as a suitable *in vivo* ferroptosis model and point to glutathione-independent actions of erastin.

## Highlights

• Drosophila can serve as an *in vivo* model of ferroptosis.
• Erastin and RSL3 shorten fly lifespan, an effect rescued by ferrostatin-1.
• In male flies erastin disrupts iron homeostasis without depleting total glutathione.
• In male flies RSL3 remodels the lipid profile.
• Flies lack an obvious xCT; the GPX4 ortholog is thioredoxin-dependent and essential.

## Graphical abstract

**Figure:**
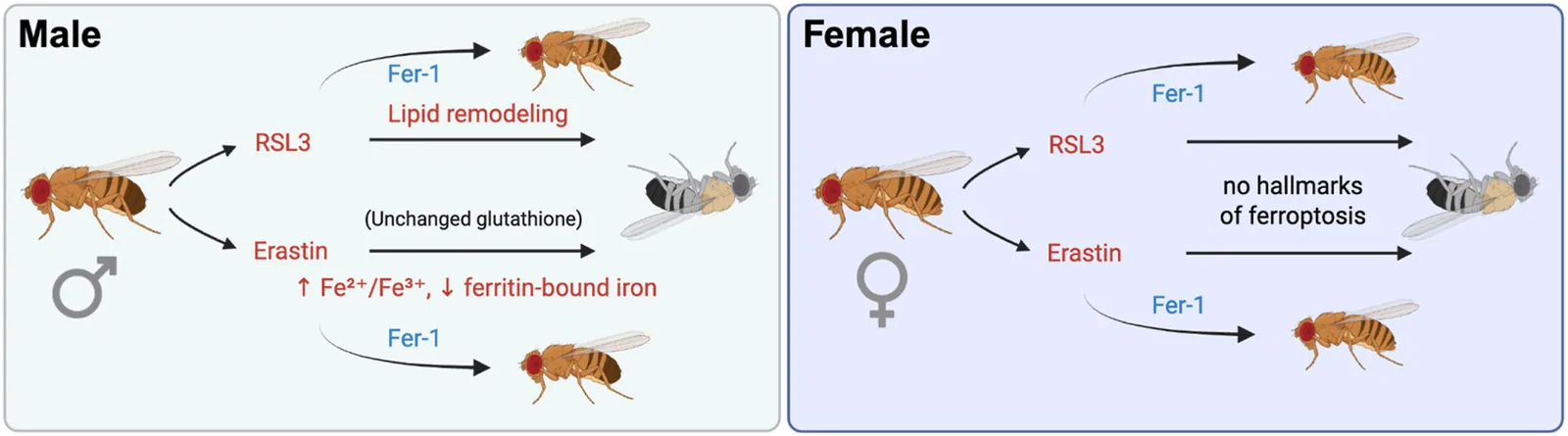

## 1 Introduction

Ferroptosis is a form of regulated cell death distinct from apoptosis, necroptosis, and autophagy-dependent cell death. It is characterized by the iron-dependent accumulation of lipid hydroperoxides, particularly in phospholipid membranes, which ultimately leads to cell death (reviewed in Refs. [1,2]). Iron plays a critical role in ferroptosis by catalyzing the Fenton reaction, which generates highly reactive hydroxyl radicals that initiate lipid peroxidation. Glutathione peroxidase 4 (GPX4) is a key regulator of ferroptosis, as it catalyzes the reduction of lipid hydroperoxides to non-toxic lipid alcohols. Inhibition of GPX4 activity or depletion of its cofactor, glutathione, can promote ferroptosis [3]. Ferroptosis suppressor protein 1 (FSP1) is a glutathione-independent ferroptosis suppressor [4].

In humans and mice, ferroptosis can be triggered by the compound erastin which is mainly thought to induce glutathione depletion through inhibition of system xc- [5], a transport system that exchanges intracellular glutamate with extracellular cystine [6]. System xc-is composed of two proteins, SLC7A11 (xCT), the light chain subunit that specifically mediates the exchange of extracellular cystine for intracellular glutamate, and SLC3A2 (4F2hc or CD98), the heavy chain subunit responsible for proper trafficking and functioning of xCT at the plasma membrane [7]. Once imported, cystine is reduced to cysteine, an indispensable building block of the tripeptide glutathione (γ-L-glutamyl-l-cysteinylglycine), the major non-protein thiol and antioxidant. Erastin has also been described to mediate ferroptosis via inhibition of voltage-dependent anion channels (VDACs) in the outer mitochondrial membrane, causing mitochondrial dysfunction, altered permeability transition, and oxidative stress [8]. Another, glutathione-independent trigger of ferroptosis is RSL3 which is usually thought to directly inhibit GPX4 [9]. Commonly used inhibitors of ferroptosis are ferrostatin-1 (Fer-1), liproxstatin-1 (Lip-1) and deferoxamine that tackle specific targets within the ferroptotic cascade [10]; Fer-1 and Lip-1 are lipophilic radical-trapping agents that prevent lipid peroxidation and deferoxamine is an iron chelator.

Most mechanistic insight into ferroptosis has come from cultured cells, while *in vivo* models remain comparatively scarce. Those that exist are largely rodent-based and rely on genetic or pharmacologic interventions. For instance, FSP1 depletion sensitized tumors to ferroptosis and suppressed growth in a xenograft model [11,12]. Other mouse models have explored the role of ferroptosis in tissue ischemia/reperfusion (I/R) injury. Loss of FSP1 or active-site mutation of GPX4 sensitized mouse kidneys to I/R-induced damage, leading to shorter survival [13]. Conversely, the ferroptosis inhibitors Fer-1 or Lip-1 alleviated symptoms in mouse models of I/R-induced cardiomyopathy [14,15], ischemic stroke [16], and intestinal I/R injury [17]. Other mouse models implicated ferroptosis in autoimmune and inflammatory disease. In psoriasis [18,19] and experimental autoimmune encephalomyelitis, a model of multiple sclerosis [20,21], ferroptosis inducers aggravated and Fer-1 or Lip-1 treatment alleviated the symptoms, whereas in a mouse model of rheumatoid arthritis, ferroptosis induction instead attenuated disease by killing pathogenic synovial fibroblasts [22]. Ferroptosis has also been modeled in genetically tractable non-mammalian organisms, including *Danio rerio* [23] and *Caenorhabditis elegans* [24,25].

*Drosophila melanogaster*, with its short life cycle, low cost, and unmatched genetic toolkit, has been proposed as a valuable model for investigating ferroptosis *in vivo.* Early work demonstrated that mitochondrial ferritin overexpression protects against erastin-induced reduction in lifespan by regulating iron homeostasis [26]. Ferritin heavy chain depletion was subsequently shown to cause accumulation of reactive oxygen species and mitochondrial defects in the developing wing, taken as evidence for ferroptotic cell death [27]. Cadmium chloride exposure was then suspected to induce ferroptosis on the basis of ferrostatin-1 rescue, with reported inhibition of Nrf2 signaling, accumulation of lipid peroxidation, impairment of the GPX4 antioxidant system, and upregulated iron metabolism [28]. Overexpression of the Parkinson's disease-related protein α-synuclein showed that increased iron and reduced glutathione exacerbated neurodegeneration and lifespan reduction [29], and age-related studies found that older flies exhibit increased iron, decreased glutathione, and increased lipid peroxidation [30]. In PINK1 mutant flies modeling Parkinson's disease, iron accumulation, lipid peroxidation, and decreased GPX activity were detected in brains, with ferrostatin-1 ameliorating pathological phenotypes [31]. Disruption of iron homeostasis was likewise central when fat-body knockdown of juvenile hormone acid methyltransferase (Jhamt) led to iron accumulation through upregulated iron importers, again with ferrostatin-1 providing protection [32]. Most recently, Davidson et al. achieved the first direct visualization of ferroptosis-like cell death in *Drosophila* embryos during acute injury inflicted by laser ablation and needle puncture wounding, based on BODIPY 581/591 C11 lipid-peroxidation imaging, mitochondrial depolarization, and rescue by ferrostatin-1 and liproxstatin-1, though they explicitly framed this as "ferroptosis-like" rather than canonical ferroptosis [33]. Together these studies show that ferroptosis-related processes operate in flies, but the evidence is heterogeneous - several infer ferroptosis mainly from ferrostatin-1 rescue, and most address a specific disease context rather than the fly as a general model. A systematic comparison of class 1 (erastin) and class 2 (RSL3) inducers across the canonical hallmarks, combined with a conservation analysis and genetic loss of the GPX4 ortholog, is still lacking.

Here we address this gap by first compiling a comprehensive inventory of conserved ferroptosis-related genes and then comparing erastin and RSL3 side by side for their effects on lifespan, glutathione, iron speciation, and lipid composition in both sexes, complemented by genetic loss of the GPX4 ortholog Gtpx and studies on the identity of system xc-light chain proteins. Our work defines how far canonical ferroptosis is conserved in *Drosophila* and establishes male *w*^*1118*^ flies as a tractable *in vivo* model.

## 2 Materials and methods

### 2.1 Fly stocks and husbandry

*w*^*1118*^, Canton-S, and Oregon-R wild-type strains were used. The heterozygous *Gtpx* knockout line carries a CRISPR-Mediated Integration Cassette (CRIMIC) inserted into the *Gtpx*/CG12013 locus [34,35] (Bloomington stock #94973), maintained over a TM3, Sb, Ser balancer. The deficiency line Df(3L)ED208 (Bloomington stock #8059) was used for complementation testing. Flies were reared on standard molasses-based food at 25°C under a 12 h:12 h light:dark cycle, with fresh food provided every 2 days, as described previously [36,37].

### 2.2 Fly treatments and lifespan

Lifespan was determined using approximately 25 flies per group and four to six groups of age-matched adults, collected post-eclosion and separated by sex. Compounds were delivered on filter paper soaked with a 5% (w/v) sucrose/water solution, an established method for oral drug administration in adult *Drosophila* [38], or mixed into standard fly food, as indicated; sucrose was used without solid food to ensure reliable ingestion of the compound. Flies were fed daily with 1 or 10 μM erastin (MedChem Express, HY-15763), 2 or 20 nM RSL3 (Biomol, Cay19288-1), 0.5 μM ferrostatin-1 (Sigma Aldrich, SML0583-5 MG), or an equivalent volume of DMSO as vehicle control. Each compound was dissolved in DMSO (Carl Roth, A994.2). Filter papers were kept adequately moistened and refreshed regularly to prevent dehydration. Mortality was recorded every 2 days, with fresh food supplied at each interval. For offspring counting, five female *Gtpx*-KO/balancer (or Df(3L)ED208/balancer) flies were crossed with three male *Gtpx*-KO/balancer flies across four replicates, and all F1 offspring were genotyped and counted.

### 2.3 GSH assay

Total glutathione was determined using the DTNB–glutathione reductase recycling assay. All steps were performed on ice, all centrifugation was performed at 4 °C. Samples were prepared from five 7-day-old flies each per replicate (male or female), treated with 1 μM or 10 μM erastin (or DMSO as vehicle control) for 7 days. Samples were homogenized in PBS/EGTA and centrifuged at 500 x g for 3 min. The supernatant was collected and deproteinized with 10% sulfosalicylic acid. Samples were incubated on ice for 10 min and centrifuged at 18.800 x g for 10 min. Subsequently, 24 μL of 1:1 triethanolamine:H_2_O was added to 280 μL of supernatant and vortexed. Then, the assay mix, consisting of 0.12 mg/mL 5,5′-dithiobis(2-nitrobenzoic acid) (DTNB), 0.33 mg/mL NADPH, and 0.5% (v/v) glutathione reductase (Sigma-Aldrich, G3664-500UN), was added quickly. Right after addition of the assay mix, absorbance was measured at 412 nm over 30 min. GSH levels were quantified against a standard curve and normalized to protein content determined by BCA assay.

### 2.4 Polymerase chain reaction

For quantitative PCR, RNA from five 7-day-old whole male flies per sample, treated with either 10 μM of erastin or DMSO as vehicle control, was extracted with the ZR RNA MiniPrep kit (Zymo Research) and 1 μg was reverse-transcribed using the High-Capacity cDNA Reverse Transcription Kit (Life Technologies). qPCR was performed with the FastStart Universal SYBR Green Master kit (Sigma-Aldrich 4913850001) on a LightCycler 480 Instrument (Roche), with 10 ng cDNA and 10 pmol of each primer per reaction under the following cycling conditions: Holding stage: 50°C, increasing to 95 °C over 2 min, then hold at 95 °C for 10 min. Cycling stage: 95°C for 15 s, 60°C for 1 min, 40 cycles. Melting curve: 95°C for 15 s, 60 °C for 1 min, 95 °C for 15 s (slope +0.3°C). Transcript levels were calculated by the 2^−ΔΔCt method [39] with Rp49 as the housekeeping gene; ΔΔCt = ΔCt(experimental) − mean ΔCt(control), ΔCt = Ct(gene of interest) − Ct(housekeeping). Primer sequences (5′→3′).

**Table:** 

Gene	Forward	Reverse
Rp49	CTGTCGCACAAATGGCGCAA	GCGCTTGTTCGATCCGTAAC
Gb	GCCATCGCTGTGACCTTTGGCG	GGGAGGAGGTCATGATGTGGACGG
Mnd	GGTTCGCTGAATGGAGCGATCTTCGC	GGTGAGCACGCCCAGGAATATCAGC
JhI-21	CCCACAAATGGATGCGCCGCG	CCCACGGATTCGGTGTAAATAAAGACTCCCG
CG1607	TGCCACCATATTCGTGACCG	CTGCTTTCCGAGTACCATGAGC
Sbm	CCTGATGAGTCGGTCTCTGG	GCAAGTCATGTATCAGCATGTCC

Primers used for quantitative PCR analysis. All primer pairs were designed to span exon-exon junctions to avoid amplification of genomic DNA.

For endpoint PCR, genomic DNA was extracted from single adult flies homogenized in 50 μL of 10 mM Tris-HCl (pH 8.0), 1 mM EDTA, 25 mM NaCl, plus 1 μL Proteinase K. 1 μL served as template, amplified with GoTaq G2 polymerase (Promega, M7845) using ACCAGAAGCCTGATAGCACCC/CCGTACTTCTCCTTTAGATCCGTCA (wildtype *Gtpx*) or GTCGCCGGTTTCAGTTTTATTTAC/TTCAGGATCATATCCAGATCCTCG (CRIMIC-cassette Kozak sequence of the *Gtpx* knockout). Products were resolved by agarose-gel electrophoresis.

### 2.5 Lipid quantification

For lipidomics, four samples each containing four 7-day-old male or female adult *w*^*1118*^ flies, treated with either 10 μM erastin, 20 nM RSL3, or DMSO as vehicle control, were processed as described previously [40]. Mass spectra were recorded on a Thermo Q-Exactive Plus spectrometer with a standard HESI-II ion source for shotgun lipidomics. Raw files were converted to mzML and analyzed with LipidXplorer v1.2.8 [41]. Absolute amounts were calculated from internal-standard intensities and all identified lipids were normalized to total lipid amount.

### 2.6 CE-ICP-MS

For iron redox speciation (ferritin-bound iron, free Fe^3+^, free Fe^2+^), five groups of 3 7-day-old male or female adult *w*^*1118*^ flies, treated with either 10 μM erastin, 20 nM RSL3, or DMSO as vehicle control, were compared by capillary electrophoresis coupled to inductively coupled plasma mass spectrometry (CE-ICP-MS), as described previously [37,42,43]. Separations used a PrinCe 706 CE system with an uncoated capillary (100 cm × 50 μm ID; CS-Chromatographie Service) at +20 kV, with sample tray and capillary maintained at 20 °C; the CE-ICP-MS interface enabled electrical connection while preventing suction flow by operating in self-aspiration mode. Electrolytes were 10% HCl (leading), 0.05 mM HCl (terminating), and 50 mM HCl (background). A NexIon 300-D ICP-DRC-MS (PerkinElmer) served as element-selective detector; ^56^Fe and ^54^Fe were measured in dynamic reaction cell (DRC) mode using ammonia as DRC gas (0.58 mL NH_3_/min), with RF power 1250 W, plasma gas 16 L Ar/min, and a nebulizer gas flow of 0.98 L Ar/min at the interface. Electropherograms were analyzed with Peakfit (Systat). To verify recovery, total iron was determined by ICP-sector field MS (ELEMENT 2, Thermo Scientific; RF power 1260 W, plasma 16 L Ar/min, auxiliary 0.85 L Ar/min, nebulizer 1.085 L Ar/min, dwell time 300 ms); quantified iron species relative to total iron ranged between 92% and 110%. Fe^2+^/Fe^3+^ and Fe-ferritin/total-Fe ratios were derived from the quantified concentrations.

### 2.7 Phylogenetic analysis of candidate fly xCT orthologs

Protein sequences for the five *Drosophila* SLC7-family light chains (gb/CG6070, mnd/CG3297, JhI-21/CG12317, CG1607, sobremesa/CG9413), human SLC7A11 (xCT) and SLC7A5 (LAT1), zebrafish SLC7A11, and human SLC1A1 (EAAT3, outgroup) were retrieved from UniProt (accessions Q9VB75, Q9Y1A7, Q7KHM4, Q9V9Y0, Q8IR48, Q9UPY5, Q01650, A0A140LGS0, and P43005, respectively). Sequences were aligned using Clustal Omega on UniProt with default parameters [44]. A maximum-likelihood phylogenetic tree was constructed from the alignment using IQ-TREE [45] with automatic substitution-model selection and 1000 ultrafast bootstrap replicates. Human SLC1A1 (EAAT3) served as an outgroup. The tree was visualized as a phylogram with branch lengths proportional to substitutions per site.

### 2.8 Cloning and overexpression of fly system xc^−^ light- and heavy-chain orthologs in HT22 cells

Codon-optimized JhI-21 was synthesized (Thermo Fisher) and cloned with a C-terminal HA tag into a PiggyBac-based vector expressing TagBFP2 from a separate promoter; TagBFP2 alone served as empty-vector control. Vectors were co-transfected with a PBase-expressing vector into HT22 cells (originally obtained from Dave Schubert, Salk Institute, San Diego/USA) using Attractene (manufacturer's protocol). After 48 h, BFP-positive cells were collected by FACS and expanded; sorting and expansion were repeated four times until ∼90% BFP positivity. A codon-optimized heavy-chain ortholog of CD98hc was synthesized (Thermo Fisher), cloned with a C-terminal FLAG tag into a PiggyBac vector co-expressing mCherry from the same promoter, and transfected into HT22 lines stably expressing *JhI-21*; transfection was validated by mCherry fluorescence.

### 2.9 Cell viability assay

HT22 viability was assessed by MTT assay. Cells were seeded at 5000/well in 96-well plates (100 μl) in complete medium. The next day, 10 μl of a 10× compound stock was added to give the indicated erastin concentrations or vehicle. After 24 h, medium was replaced with MTT (0.5 mg/ml in HBSS with Ca^2+^/Mg^2+^) and incubated 1 h at 37°C. Formazan crystals were dissolved in 100 μl DMSO and absorbance read at 590 nm (Tecan NanoQuant Infinite M200 Pro).

### 2.10 Immunoblotting

Cells were lysed in RIPA buffer with protease/phosphatase inhibitors (Roche), centrifuged at 19,000 × g for 15 min at 4 °C, and supernatants collected. Proteins were denatured in Laemmli buffer with 50 mM DTT (95 °C, 5 min), separated by SDS-PAGE, and transferred to nitrocellulose. Membranes were blocked in 5% milk/TBST (1 h, RT), incubated overnight at 4 °C with primary antibodies (mouse anti-actin JLA20, DSHB, AB_528068, 1:500; mouse anti-HA 16B12, BioLegend, 1:1000), then with fluorescent secondary antibodies (Cell Signaling Technology, 1:10,000; 1 h, RT). Signals were detected on an Odyssey system (LI-COR) and quantified with Image Studio Lite, normalized to loading control and average blot signal.

### 2.11 Statistics

Data were analyzed with GraphPad Prism. For each comparison, we first assessed normality using the Shapiro-Wilk test. When normality was met, we used parametric tests (unpaired *t*-test, or one-way ANOVA with Dunnett's multiple-comparisons test comparing each treatment group against vehicle); otherwise, we used non-parametric tests (Mann-Whitney, or Kruskal-Wallis with Dunn's multiple-comparisons test). We compared lifespan/survival curves by the log-rank (Mantel-Cox) test together with the Gehan-Breslow-Wilcoxon test, considering a difference significant only when both tests concurred. We corrected lipidomics data for multiple comparisons using the two-stage step-up method of Benjamini, Krieger and Yekutieli at a 1% false-discovery rate [46]. We assessed deviations from expected Mendelian ratios by a binomial test. Data are presented as box-and-whisker plots (box, 25th–75th percentile; whiskers, min–max) showing all individual data points. n denotes biological replicates. We assumed statistical significance at *p* < 0.05.

## 3 Results

### 3.1 Presence and absence of proteins involved in ferroptosis in *Drosophila melanogaster*

Before assessing whether ferroptosis can be pharmacologically induced in flies, we asked how much of the mammalian ferroptosis machinery is conserved in *Drosophila melanogaster*. We compiled the core human ferroptosis-related proteins and bioinformatically identified their fly orthologs (Table 1). The majority are conserved, but several components are either absent or functionally divergent, and these differences cluster in a few modules summarized in Fig. 1: iron handling, cystine import, the glutathione/GPX4 axis, and the parallel radical-trapping systems.

**Table 1:** Fly orthologs of human proteins involved in ferroptosis.

Human gene	Fly ortholog (Symbol/Name/CG number)	Similarity	Identity	Gap
Acyl-CoA synthetase long chain family member 4 (ACSL4)	Acsl/Acyl-CoA synthetase long-chain/CG8732	63	47	6
Lysophosphatidylcholine acyltransferase 3 (LPCAT3)	nes/nessy/CG9655	57	37	5
Membrane bound O-acyltransferase domain containing 1 (MBOAT1)	oys/oysgedart/CG18445	57	41	2
Membrane bound O-acyltransferase domain containing 2 (MBOAT2)	oys/oysgedart/CG18445	56	40	4
Arachidonate lipoxygenases (ALOXs)	No high/moderate DIOPT v10 ortholog			
Cytochrome P450 oxidoreductase (POR)	Cpr/Cytochrome P450 reductase/CG11567	74	56	2
Transferrin (TF)	Tsf2/Transferrin 2/CG10620	45	31	23
	Tsf1/Transferrin 1/CG6186	38	23	25
	Tsf3/Transferrin 3/CG3666	36	22	27
Transferrin receptor (TFRC/TFR1)	CG15522/-/CG15522	40	23	13
Solute carrier family 11 member 2 (SLC11A2/DMT1)	Mvl/Malvolio/CG3671	74	59	4
Nuclear receptor coactivator 4 (NCOA4)	No high/moderate DIOPT v10 ortholog			
Ferritin light chain (FTL)	Fer3HCH/Ferritin 3 heavy chain homologue/CG4349	62	36	2
	Fer1HCH/Ferritin 1 heavy chain homologue/CG2216	51	27	7
	Fer2LCH/Ferritin 2 light chain homologue/CG1469	43	24	6
Ferritin mitochondrial (FTMT)	Fer3HCH/Ferritin 3 heavy chain homologue/CG4349	62	45	2
	Fer1HCH/Ferritin 1 heavy chain homologue/CG2216	48	31	4
	Fer2LCH/Ferritin 2 light chain homologue/CG1469	44	26	11
Solute carrier family 7 member 11 (SLC7A11)	CG1607/-/CG1607	33	19	36
	JhI-21/Juvenile hormone Inducible-21/CG12317	38	18	24
	gb/genderblind/CG6070	64	42	5
	mnd/minidiscs/CG3297	68	46	0
	sbm/sobremesa/CG9413	60	39	5
Glutamate-cysteine ligase catalytic subunit (GCLC)	Gclc/Glutamate-cysteine ligase catalytic subunit/CG2259	34	22	26
Glutamate-cysteine ligase modifier subunit (GCLM)	Gclm/Glutamate-cysteine ligase modifier subunit/CG4919	49	29	16
Glutathione synthetase (GSS)	Gss2/Glutathione synthetase 2/CG32495	52	37	15
	Gss1/Glutathione synthetase 1/CG6835	52	37	15
Glutathione peroxidase 4 (GPX4)	Gtpx/Glutathione peroxidase homologue with thioredoxin peroxidase activity/CG12013	54	37	12
	Gpxl/Glutathione peroxidase-like/CG15116	55	38	6
Glutathione-disulfide reductase (GSR/GR)	Trxr1/Thioredoxin reductase 1/CG2151	53	36	6
	Trxr2/Thioredoxin reductase 2/CG11401	53	35	9
Thioredoxin reductase 2 (TXNRD2)	Trxr1/Thioredoxin reductase 1/CG2151	67	53	5
	Trxr2/Thioredoxin reductase 2/CG11401	70	55	1
Thioredoxin (TXN)	Trx2/Thioredoxin 2/CG31884	66	51	0
	TrxT/Thioredoxin T/CG3315	31	20	38
	dhd/deadhead/CG4193	51	29	5
	CG13473/-/CG13473	64	39	1
Ferroptosis suppressor protein 1 (AIFM2/FSP1)	No high/moderate DIOPT v10 ortholog			
GTP cyclohydrolase 1 (GCH1)	Pu/Punch/CG9441	71	60	10
Dihydroorotate dehydrogenase (DHODH)	Dhod/Dihydroorotate dehydrogenase/CG9741	64	47	3
NFE2-like bZIP transcription factor 2 (NFE2L2/NRF2)	cnc/cap-n-collar/CG43286	42	33	33
Activating transcription factor 4 (ATF4)	crc/cryptocephal/CG8669	36	23	27
Apurinic/apyrimidinic endodeoxyribonuclease 1 (APEX1/Ref-1)	Rrp1/Recombination repair protein 1/CG3178	60	47	15

**Fig. 1:**
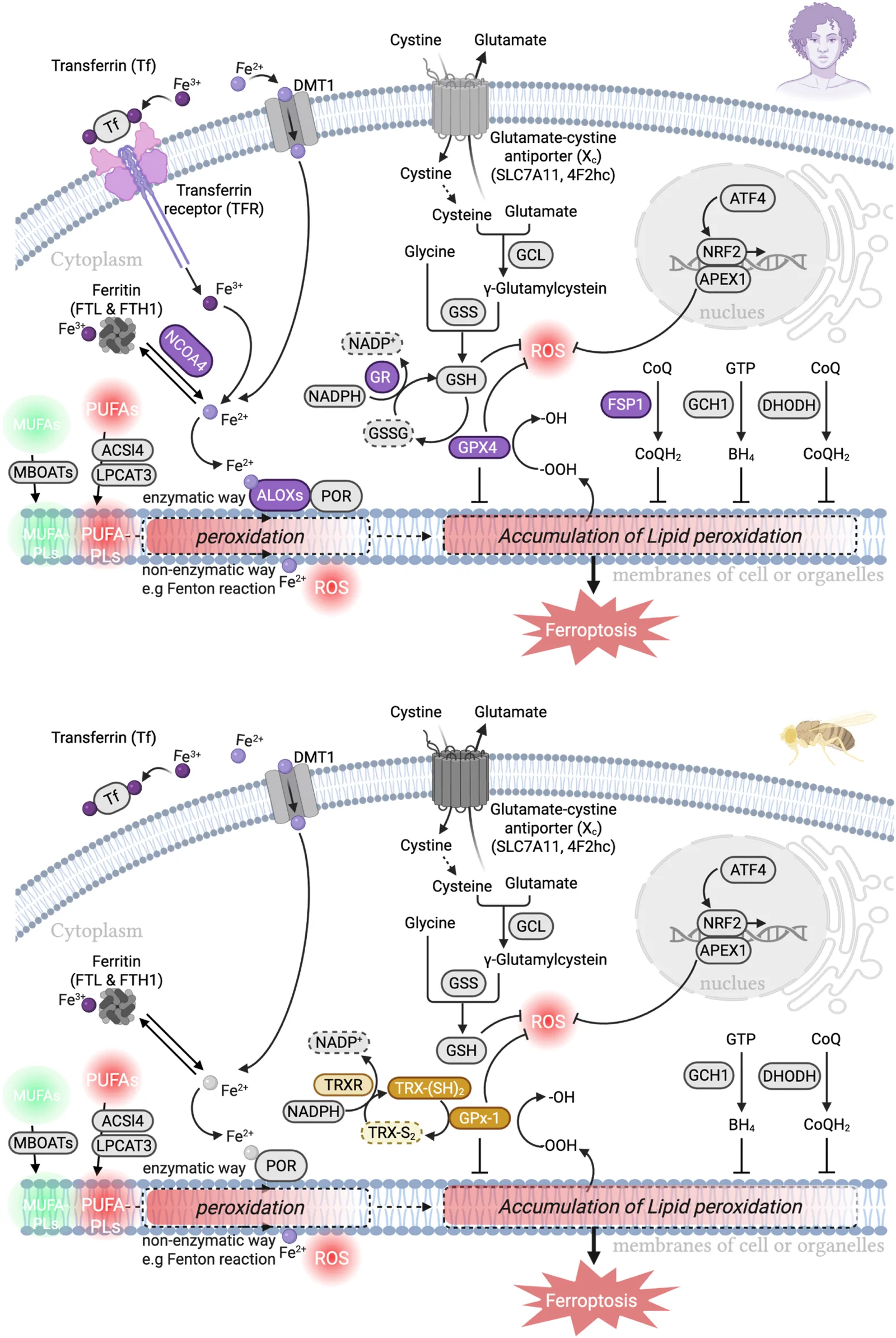
**Conservation and divergence of the ferroptosis machinery between humans and *Drosophila melanogaster.*** Ferroptosis-related proteins and metabolites in humans and *Drosophila melanogaster*. Pathways conserved in both species are shown in gray. Proteins in purple have no identifiable fly ortholog; proteins in yellow are conserved but functionally divergent in the fly. The dashed glutamate-cystine antiporter (system xc^−^) denotes its uncertain conservation in *Drosophila*. Fly ferritin (Fer1HCH/Fer2LCH) is predominantly secretory and hemolymph-associated rather than cytosolic. Orthologs were identified through FlyBase using the DRSC Integrative Ortholog Prediction Tool (DIOPT) version 10; scores are listed in Table 1.

The first divergence concerns iron uptake and storage. In humans, iron is imported both through the transferrin/transferrin-receptor pathway and through the divalent metal transporter 1 (DMT1) following reduction of ferric to ferrous iron. *Drosophila* relies essentially on the DMT1 ortholog Malvolio (Mvl) and lacks a transferrin receptor, so it has no transferrin-receptor-mediated uptake route [47]. Flies do encode transferrins, but these circulate in the hemolymph and their contribution to cellular iron import remains uncertain [47]. For iron storage, both lineages use ferritin, but the fly protein differs in composition and localization: it is a heteropolymer of the heavy-chain homologue Fer1HCH and the light-chain homologue Fer2LCH (with the mitochondrial homologue Fer3HCH corresponding to human FTMT), and it is predominantly routed through the secretory pathway and the hemolymph rather than residing in the cytosol [47]. In addition, *Drosophila* lacks an ortholog of NCOA4, the receptor that in mammals targets ferritin for autophagic degradation (ferritinophagy) to release stored iron.

The pro-ferroptotic lipid machinery, in contrast, is well conserved. The enzymes that generate the oxidation-prone membrane substrates of ferroptosis, acyl-CoA synthetase long-chain family member 4 (ACSL4) [48] and lysophosphatidylcholine acyltransferase 3 (LPCAT3) [49], together activate and incorporate polyunsaturated fatty acids into membrane phospholipids and thereby set ferroptosis sensitivity in mammalian cells. Both have clear fly orthologs (Acsl/CG8732 and Nes/CG9655; Table 1). Of the two membrane-bound O-acyltransferases recently shown to suppress ferroptosis, only MBOAT2 is conserved in the fly (oys/CG18445), whereas MBOAT1 has no ortholog. At the level of lipid peroxidation itself, humans use both arachidonate lipoxygenases (ALOXs) and cytochrome P450 oxidoreductase (POR), whereas *Drosophila* possesses only POR (Cpr/CG11567) and no ALOX ortholog.

The most fundamental divergence lies in the glutathione/GPX4 axis. In humans, glutathione peroxidase 4 (GPX4) reduces lipid hydroperoxides to the corresponding alcohols using reduced glutathione (GSH) as the electron donor, and the oxidized glutathione (GSSG) produced is recycled to GSH by glutathione reductase (GR) at the expense of NADPH. *Drosophila* differs at two points. First, it expresses no genuine glutathione reductase; instead, GSSG is re-reduced by the thioredoxin system, a defining and unusual feature of insect antioxidant defense [50]. Second, the closest GPX4 ortholog, *Gtpx* (CG12013), lacks the catalytic selenocysteine and behaves as a thioredoxin peroxidase rather than a glutathione peroxidase, drawing its reducing equivalents from the thioredoxin reductase-thioredoxin system rather than from GSH [51]. Notably, fly TrxR (Trxr-1, encoding both cytosolic and mitochondrial isoforms) is itself a non-selenocysteine protein and indispensable for viability [52]. The fly therefore retains the capacity to detoxify lipid hydroperoxides but does so through a thioredoxin-, rather than glutathione-, dependent route which is an important consideration when interpreting glutathione-targeting compounds such as erastin in this organism.

A related uncertainty concerns the entry point that erastin targets in mammals: the cystine/glutamate antiporter system xc^−^. In humans this transporter is a heterodimer of the light chain SLC7A11 (xCT) and the heavy chain SLC3A2 (CD98/4F2hc) [7]. The fly genome encodes a single CD98 heavy-chain homologue (CG2791) and five SLC7-family light chains – genderblind (gb/CG6070), minidiscs (mnd/CG3297), JhI-21 (CG12317), CG1607, and sobremesa (CG9413) – all of which retain the conserved interchain cysteine of the heterodimeric amino-acid transporters [53]. One of these, gb, was originally proposed to act as a cystine/glutamate antiporter (an xCT) on the basis of a reduced extracellular-glutamate phenotype in mutants [54]. Subsequent functional characterization, however, has assigned these light chains predominantly to system L, the sodium-independent exchange of large neutral amino acids rather than to cystine/glutamate transport: mnd behaves as a LAT1-like transporter [55], JhI-21 mediates system-L transport when paired with CD98 [53], and sobremesa is likewise a glial system-L LAT [56]. Whether any fly light chain constitutes a bona fide, erastin-sensitive system xc^−^ therefore remains unresolved.

Finally, the glutathione-independent, radical-trapping defenses are only partially conserved. *Drosophila* lacks an ortholog of ferroptosis suppressor protein 1 (FSP1/AIFM2), the NAD(P)H-dependent CoQ oxidoreductase that constitutes a major GPX4-independent inhibitor of ferroptosis in mammals [4]. It does, however, retain the two more recently described parallel systems: dihydroorotate dehydrogenase (DHODH; Dhod/CG9741), which reduces ubiquinone in the inner mitochondrial membrane [57], and GTP cyclohydrolase 1 (GCH1; Pu/Punch/CG9441), the rate-limiting enzyme for tetrahydrobiopterin synthesis that acts as an endogenous radical-trapping antioxidant [58]. The upstream stress-response and transcriptional regulators (NRF2/cnc, ATF4/crc, APEX1/Rrp1) are likewise conserved (Table 1).

Together, this inventory shows that *Drosophila* possesses the substrate-generating and iron-dependent core of the ferroptosis pathway but diverges in two respects most relevant to the pharmacology used here: a functional, erastin-sensitive system xc^−^ has not been identified and the GPX4-related peroxide-reduction system is thioredoxin-dependent rather than glutathione-dependent.

### 3.2 Erastin and RSL3 dose-dependently reduce the lifespan of male and female *Drosophila melanogaster* flies

To study ferroptosis in flies, we fed flies with erastin and RSL3 dissolved in sucrose and determined their survival. The diluent sucrose served as control. We did not include an FSP1 inhibitor because FSP1 is not conserved in flies (Fig. 1). Both erastin (Fig. 2A) and RSL3 (Fig. 2B) dose-dependently shortened the lifespan of male and female *w*^*1118*^ flies, the most commonly used genetic background in fly research for generating genetically-modified fly strains. Concomitant treatment with the ferroptosis inhibitor ferrostatin-1 abolished the lifespan reduction conferred by both erastin and RSL3 in both sexes (Fig. 2A and B), establishing that the lethality is attributable to ferroptosis rather than to nonspecific toxicity. Similar results were obtained in the independent wild-type backgrounds Canton-S and Oregon-R (Supplemental Fig. 1). Female flies in general lived longer compared to males. To rule out confounding effects introduced by protein restriction of flies fed only sucrose, we repeated these experiments with erastin and RSL3 added to normal food. Under these conditions, female flies were protected against erastin (Fig. 2A) but not against RSL3 (Fig. 2B); males remained susceptible to both.

**Fig. 2:**
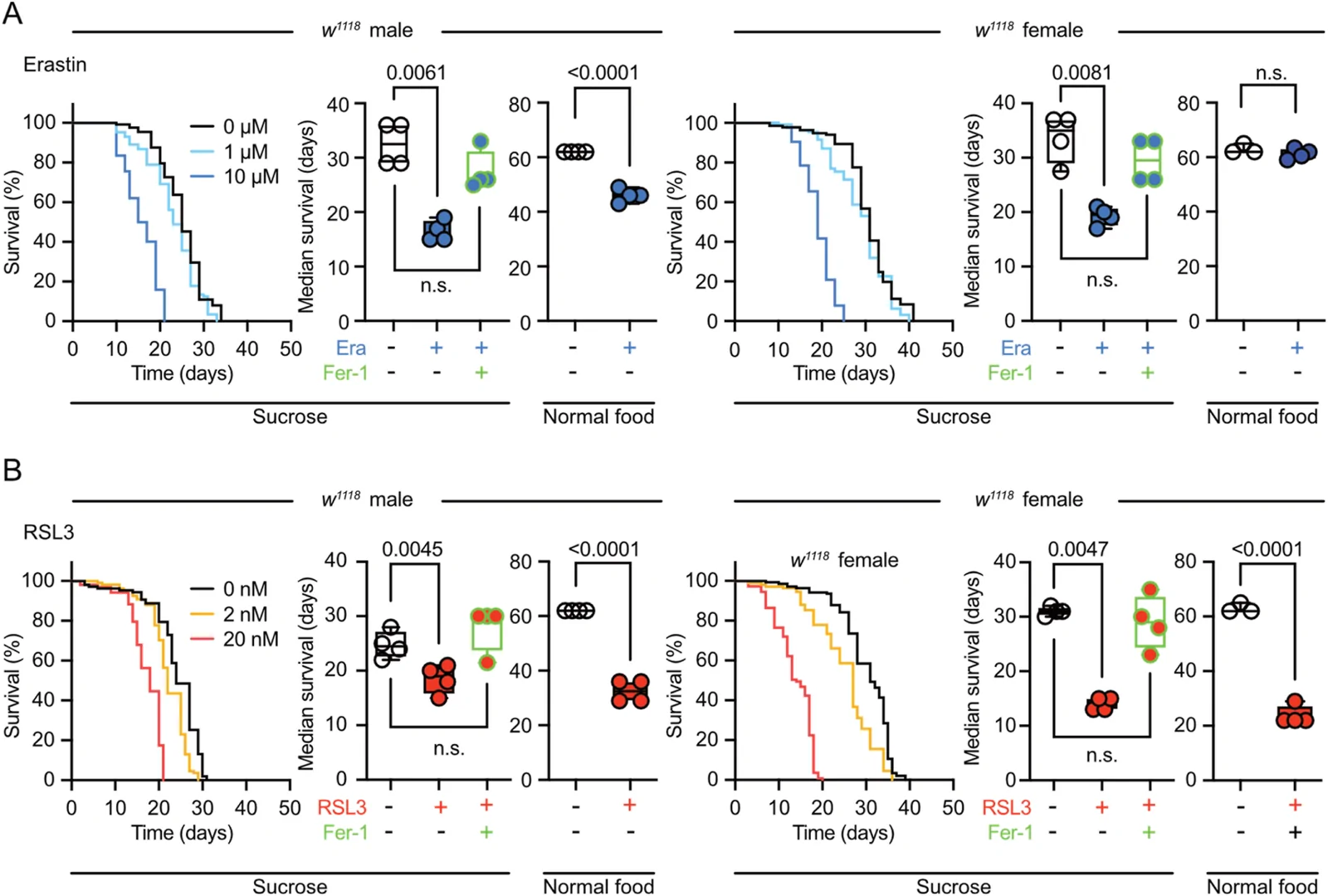
**Erastin and RSL3 dose-dependently reduce the lifespan of male and female *Drosophila melanogaster* flies.** Lifespan and median survival of male and female *w*^*1118*^ flies treated with sucrose-based or normal food containing **A)** erastin or **B)** RSL3. For median survival analysis flies were either kept on a sucrose-based diet supplemented with 10 μM erastin or 20 nM RSL3, each alone or in combination with 500 nM ferrostatin-1 (Fer-1) or on a normal food diet supplemented with 10 μM erastin or 20 nM RSL3 (DMSO served as vehicle). n = 120 per genotype per sex per dose. Box and whisker plots have the box extending from the 25th to the 75th percentile and the whiskers from the minimum to the maximum value depicting all individual data points. Statistical analysis was done using the non-parametric Kruskal-Wallis test (sucrose) and *t*-test (normal food), *p* values are indicated. n.s., non-significant.

Together these data show that the combination of sucrose feeding and erastin or RSL3 triggers a ferrostatin-sensitive form of lethality consistent with ferroptosis in both sexes, while normal food protects females against erastin. To test whether the sucrose-induced, ferrostatin-sensitive lethality is mechanistically ferroptosis, we examined its canonical biochemical hallmarks (glutathione, iron speciation, and lipid composition) in male and female flies.

### 3.3 Male but not female *w*^*1118*^ flies exhibit hallmarks of ferroptosis

To clarify whether the reduction in lifespan is accompanied by hallmarks of ferroptosis, we next studied total glutathione, iron speciation, and lipid composition in male and female *w*^*1118*^ flies treated with erastin and RSL3 under the same conditions used for the survival assays. Female (virgin and mated) flies had much higher levels of total glutathione (GSH) than male flies, but neither sex displayed any significant changes in total GSH levels after treatment with erastin for seven days; RSL3 was not assessed as it acts downstream of glutathione supply (Fig. 3A). This is in line with the reduced reliance of flies on GSH as reductant [51]. Using capillary electrophoresis coupled to inductively coupled plasma mass spectrometry (CE-ICP-MS), we next investigated changes in iron homeostasis. Here, only male flies treated with erastin demonstrated an increase in the pro-oxidative Fe^2+^/Fe^3+^ ratio (Fig. 3B) and a reduction in ferritin-bound iron (Fig. 3C). RSL3 had no discernible effect. Lipidomic profiling of the same conditions revealed a similar sex-specific pattern. To avoid multiple testing artifacts, we applied the two-stage step-up method of Benjamini, Krieger and Yekutieli at a 1% false discovery rate (FDR) [46]. This uncovered that again only male flies exhibited statistically significant changes, here in response to RSL3. Phosphatidylcholine (PC) and phosphatidylethanolamine (PE) lipids were upregulated, while ceramide phosphoethanolamine (CerPE), lysophosphatidylcholine (LPC), and ether-linked PC-O and PE-O species were downregulated (Fig. 3D and E). This pattern is in line with peroxidation-driven membrane remodeling rather than a bulk lipid shift, since the selectively depleted classes (ether-linked PC-O/PE-O and lyso-species) are precisely those most susceptible to peroxidative loss, whereas bulk PC and PE are elevated [59].

**Fig. 3:**
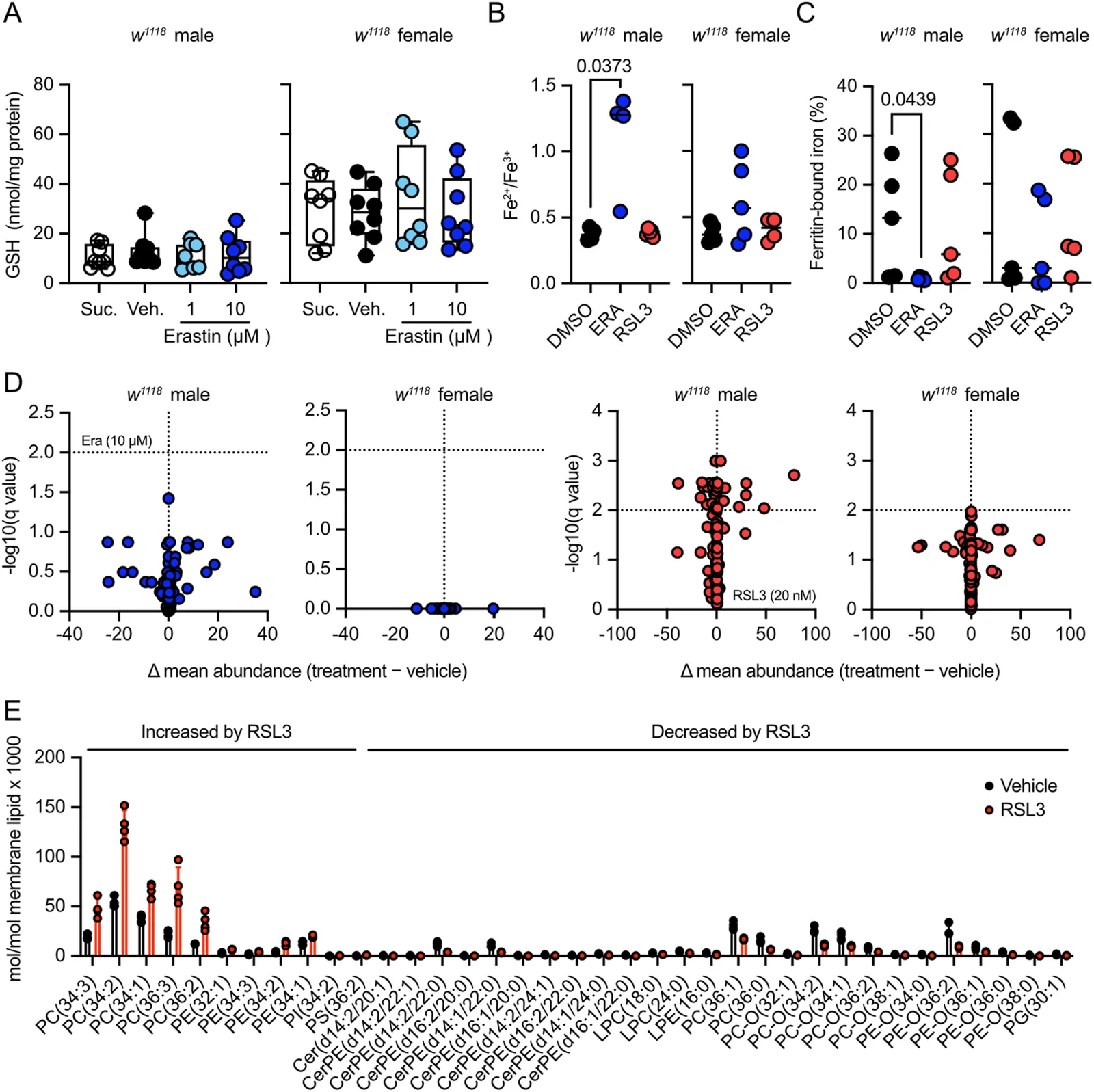
**Male but not female *w*^*1118*^ flies exhibit hallmarks of ferroptosis. A)** GSH levels of *w*^*1118*^ flies. 4 biological replicates were tested per genotype per sex. Data are presented as box and whisker plots with the box extending from the 25th to the 75th percentile and the whiskers from the minimum to the maximum value depicting all individual data points consisting of the mean value of three technical replicates of a sample of five flies. **B)** Quantification of the Fe^2+^/Fe^3+^ ratio and **C)** ferritin-bound iron levels using CE-ICP-MS. 4 biological replicates were tested per genotype per sex. Only male flies upon erastin treatment showed significant differences. **D)** Volcano plots of changes in lipid species in flies treated with erastin or RSL3. The x-axis (Δ mean abundance) shows the difference in mean normalized abundance between treatment- and vehicle-treated flies; positive values indicate species increased by treatment. Individual lipid species with a false discovery rate of 1% after correcting for multiple testing are shown above the dashed line. Only male flies upon RSL3 treatment show significant differences. **E)** Changes in lipid species abundance in male flies treated with RSL3 that were statistically significant in D. Statistical analysis was done using one-way ANOVA (A) or the non-parametric Kruskal-Wallis test (B and C), *p* values are indicated.

These findings demonstrate that male *w*^*1118*^ flies exhibit classic biochemical hallmarks of ferroptosis (disrupted iron homeostasis and altered lipid metabolism), while female flies show resistance to these molecular changes despite the reduced survival on sucrose seen in Fig. 2. Together this establishes male *w*^*1118*^ flies as a suitable model for studying ferroptosis.

### 3.4 The fly ortholog of GPX4 is indispensable for life

*Gtpx* is the most probable ortholog of GPX4 [51] (Table 1) and is ubiquitously expressed in *Drosophila* according to mRNA-Seq data [60]. To assess the consequences of *Gtpx* loss of function, we used a heterozygous *Gtpx* knockout line carrying a CRISPR-Mediated Integration Cassette (CRIMIC) [34,35] inserted into the genomic locus (Fig. 4A/B). To test whether homozygous *Gtpx* loss is viable, we crossed *Gtpx*-KO/TM3,ser,sb flies with each other. According to Mendelian inheritance, the expected offspring would be 1/3 *Gtpx*-KO/*Gtpx*-KO and 2/3 *Gtpx*-KO/TM3,ser,sb (TM3,ser,sb/TM3,ser,sb being lethal). However, we only observed *Gtpx*-KO/TM3,ser,sb offspring, indicating that homozygous *Gtpx* knockout is lethal (Fig. 4C). This lethality could not be rescued by culturing the flies on standard food supplemented with ferrostatin-1 (Fig. 4C), paralleling the constitutive embryonic lethality of mouse ubiquitous GPX4 knockouts, which is likewise refractory to pharmacological rescue [3].

**Fig. 4:**
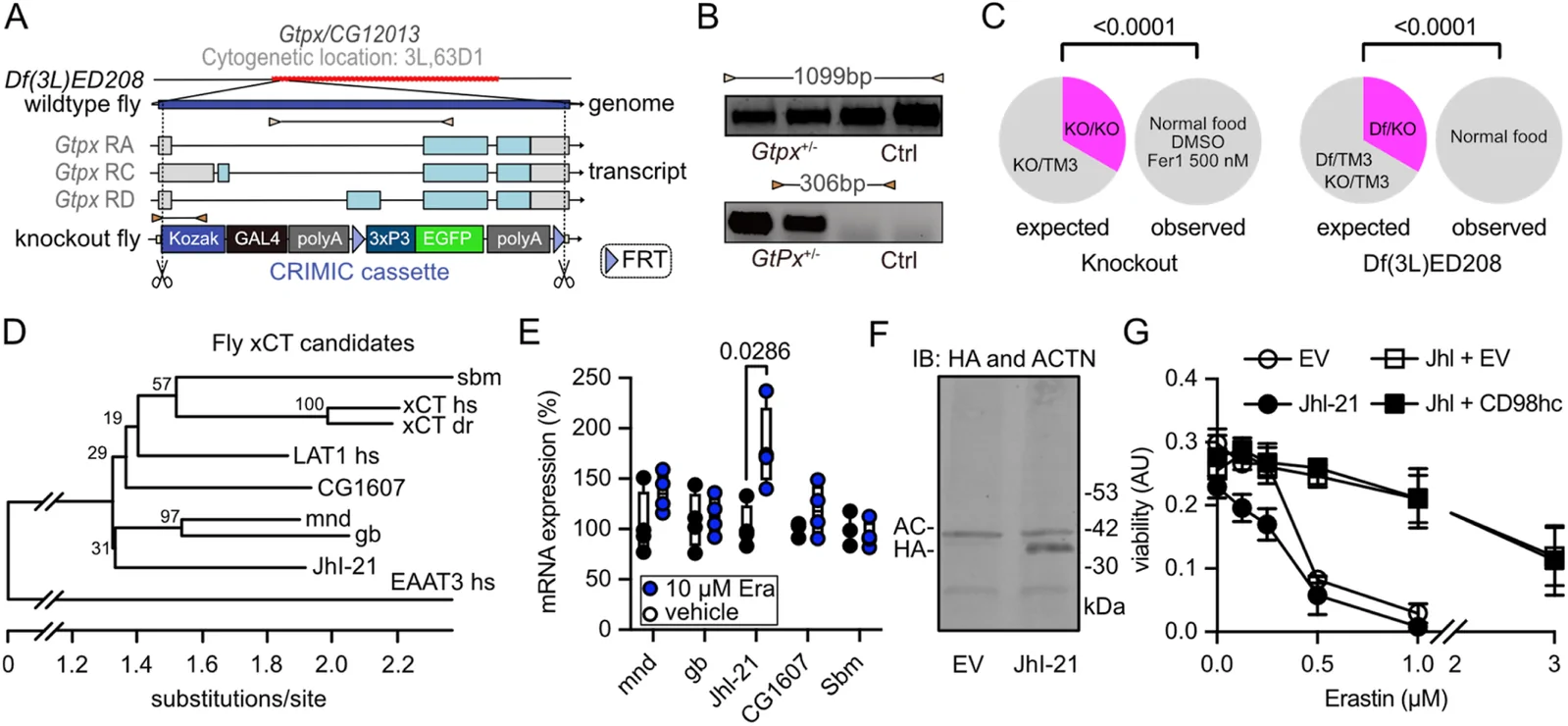
**The fly ortholog of GPX4 is indispensable for life. A)** Genomic organization of *Gtpx*/CG12013. Exons are represented by light gray boxes and coding sequences by solid blue boxes. In the *Gtpx* knockout (KO) fly, the complete gene has been replaced by the CRIMIC cassette as indicated. The deficiency line Df(3L)ED208, represented by the red dashed line, deletes the entire *Gtpx* locus. **B)** Verification of *Gtpx* KO via PCR using the primers indicated by triangles in A and B. **C)** The progeny from matings between *Gtpx* KO/TM3,ser,sb flies were expected to follow a 1:2 Mendelian ratio of *Gtpx* KO homozygotes to *Gtpx* KO/TM3,ser,sb heterozygotes, with the TM3 balancer chromosome preventing homozygosity. Only *Gtpx* KO/TM3, ser, sb offspring were observed indicating that homozygous *Gtpx* knockout is lethal. This lethality could not be mitigated by supplementing the flies' diet with 0.5 μM ferrostatin-1. DMSO served as vehicle control. Similarly, the cross between Df(3L)ED208/TM3 and *Gtpx-*KO/TM3 flies did not produce Df/KO progeny; this confirms that loss of *Gtpx* function is lethal. Statistical analysis in C was done using the binomial test. **D)** Phylogenetic tree of candidate xCT fly orthologs. No putative ortholog clusters with the vertebrate xCT clade. **E)** qPCR analysis of fly xCT orthologs in male flies normalized to rp49 with or without supplementing the flies' diet with erastin (10 μM, 7 days). DMSO served as vehicle control. Only *JhI-*21 mRNA is statistically increased upon erastin treatment quantified by the Mann-Whitney test. **F)** Immunoblot of murine HT22 cells stably transfected with HA-tagged JhI-21, or empty vector (EV). Actin (Ac) served as loading control. Expression of the HA-tag is indicated. **G)** Cell viability (MTT) of HT22 cells from F) treated with increasing concentrations of erastin for 24 h (0–1 μM, left), and of the same lines additionally co-expressing the codon-optimized fly heavy-chain ortholog CD98hc (0–3 μM erastin, right). Neither JhI-21 alone nor co-expression with CD98hc protected against erastin-induced death.

To assess whether the lethality is caused by loss of *Gtpx* rather than an unrelated mutation on the knockout chromosome, we performed a complementation test using the deficiency line Df(3L)ED208, which deletes the *Gtpx* locus and additional surrounding regions spanning approximately 650 kb (Fig. 4A). Df(3L)ED208/TM3,ser,sb flies were crossed to *Gtpx*-KO/TM3,ser,sb flies. The expected offspring would be 1/3 Df(3L)ED208/*Gtpx*-KO and 2/3 *Gtpx*-KO/TM3,ser,sb or Df(3L)ED208/TM3,ser,sb. Again, no Df(3L)ED208/*Gtpx*-KO animals were found (Fig. 4C). The failure of the *Gtpx*-KO allele to complement the deficiency argues against the lethality being due to an unrelated second-site mutation elsewhere in the genome, since such a mutation would not be removed by the deficiency and should therefore still be complemented. As we did not outcross the CRIMIC line or generate a rescue construct, we cannot formally exclude a second, independent mutation on the *Gtpx*-KO chromosome that happens to also lie within the ∼650 kb interval deleted by Df(3L)ED208. Nonetheless, together with the specificity of the CRISPR-mediated insertion into the *Gtpx* locus itself, these results are best explained by *Gtpx* being the gene responsible for the observed lethality.

The inability of dietary ferrostatin-1 to rescue is consistent with an essential requirement for Gtpx before the onset of larval feeding, since *Drosophila* embryos do not feed and orally supplied compounds only reach the animal after first-instar hatching. This places the lethal window during embryonic or very early larval development, alternatively ferrostatin-1 does not protect against Gtpx knockout. Together these results establish that Gtpx is required for viability in *Drosophila*, consistent with the essential role of its mammalian counterpart GPX4.

### 3.5 The xCT candidate JhI-21 does not confer protection against erastin in ferroptosis-sensitive mouse cells

Identifying the *Drosophila* ortholog of xCT (SLC7A11) was, however, less straightforward, because no undisputable ortholog exists in the fly genome. As a first approach to identify the most probable ortholog, we assessed the phylogenetic relationships of the five potential fly light chains to vertebrate xCT. We constructed a maximum-likelihood phylogenetic tree including the five *Drosophila* proteins, human SLC7A11 (xCT) and SLC7A5 (LAT1) as canonical references for the xc^−^ and system-L branches of the SLC7 family, and zebrafish SLC7A11 as the earliest unambiguous xCT ortholog. Human EAAT3 (SLC1A1), a structurally distinct glutamate transporter of the unrelated SLC1 family, served as an outgroup and rooted the tree on a long branch clearly separated from all SLC7 sequences (Fig. 4D). The vertebrate xCT clade (human and zebrafish SLC7A11) was strongly supported and did not include any *Drosophila* sequence or human LAT1. Among the fly light chains, mnd and gb formed a well-supported pair, consistent with paralogy, with JhI-21 branching as their most likely sister. CG1607 and sobremesa branched separately from this pair; sobremesa grouped weakly with the vertebrate xCT clade, and LAT1 in turn branched with this sobremesa/xCT group, but neither of these deeper nodes reached the support needed to interpret them biologically. No fly light chain therefore forms a robustly supported clade with vertebrate xCTs.

In mammalian cells, xCT mRNA and function are strongly upregulated in response to chronic system xc^−^ inhibition [61]. We therefore asked whether any of the five candidate light chains is transcriptionally induced by chronic erastin treatment (10 μM, 7 days) using quantitative PCR. Of the five, only *JhI-*21 mRNA was significantly increased by erastin (Fig. 4E), while *mnd*, *gb*, CG1607, and *sbm* showed no consistent change. This erastin-responsiveness prompted us to prioritize JhI-21 for functional testing. We attempted to induce ferroptosis directly in *Drosophila* cell lines (Schneider 2 and S2) but could not kill them with erastin or RSL3 at concentrations effective in mammalian cells, precluding a fly-intrinsic functional assay. We therefore stably expressed codon-optimized, HA-tagged JhI-21 in murine HT22 cells, a standard mammalian ferroptosis model in which overexpression of human xCT itself is protective against ferroptosis [61], and confirmed protein expression by immunoblotting (Fig. 4F). *JhI-21*-expressing cells were not protected against erastin; if anything, they appeared more susceptible than empty-vector controls (Fig. 4G). Since JhI-21 requires co-expression with the fly heavy chain CD98/CG2791 for system-L transport activity (system xc^−^ activity was not tested there) in *Xenopus* oocytes [53], we also co-transfected the codon-optimized fly heavy-chain ortholog of CD98hc into the JhI-21 line. Both JhI-21+CD98hc and JhI-21+EV cells showed a similar shift toward greater erastin resistance after transfection, indicating a nonspecific transfection effect rather than CD98hc-dependent rescue. Together, these results show that JhI-21, alone or with its fly heavy-chain partner, does not reconstitute protective xc^−^ activity in a mammalian cellular context.

## 4 Discussion

Our findings establish that *Drosophila melanogaster* can serve as an *in vivo* model for studying ferroptosis, while revealing important mechanistic differences from mammalian systems. Erastin and RSL3 dose-dependently shortened lifespan, and this lethality was rescued by ferrostatin-1 in both sexes, providing strong pharmacological evidence for ferroptosis. The two inducers left complementary biochemical signatures, each consistent with its mechanism of action: erastin, which acts upstream on iron and cystine handling, selectively increased the pro-oxidative Fe^2+^/Fe^3+^ ratio and reduced ferritin-bound iron in male flies, while RSL3, which acts downstream at the level of lipid peroxidation, selectively remodeled the membrane lipidome, elevating PC and PE species and depleting the peroxidation-prone ether lipids. Although these changes are consistent with ferroptosis-associated lipid remodeling, our lipidomic analysis did not directly measure oxidized phospholipid species. Together with Fer-1 rescue, the iron and lipid changes support an iron- and lipid-dependent ferroptotic phenotype. That each compound produced the hallmark expected from its target, iron dysregulation for erastin and altered lipid composition for RSL3, supports the iron-dependent, lipid-peroxidation-driven nature of this cell death and satisfies key criteria for ferroptosis. Strikingly, however, both signatures were confined to male flies, even though females also succumbed to the compounds under the sensitizing sucrose conditions.

This sex-specific pattern, biochemical hallmarks of ferroptosis in males but not in females despite comparable lethality, partially mirrors findings in mammalian systems. Female mice are protected against kidney ferroptosis through NRF2 pathway activation [62], and sex-dependent regulation of MBOAT family members influences ferroptosis susceptibility [63]. Of these, only MBOAT2 has a fly ortholog (Fig. 1 and Table 1), and the mechanisms underlying the female resistance we observe remain to be determined. Female flies were, in addition, protected against erastin-induced lethality on normal food (Fig. 2), a diet-dependent resistance whose relationship to the absence of biochemical hallmarks is unclear and was not pursued here. The occurrence of sex-dependent ferroptosis phenotypes in both flies and mammals suggests that biological sex may represent an evolutionarily relevant modifier of ferroptosis susceptibility, and makes *Drosophila* an attractive model for the genetic dissection of these pathways.

A striking difference from mammalian ferroptosis is the absence of detectable changes in total glutathione following erastin treatment. In mammalian cells, erastin lowers total glutathione by inhibiting system xc^−^, blocking cystine import and thereby limiting cysteine available for glutathione synthesis [61,64]. The unchanged total glutathione pool in erastin-treated flies may reflect the distinct organization of the fly antioxidant network: *Drosophila* lacks glutathione reductase, and its GPX4 ortholog Gtpx is a non-selenocysteine, thioredoxin-dependent peroxidase that draws reducing equivalents from thioredoxin reductase rather than from glutathione [51]. We cannot fully exclude that compensatory changes in glutathione synthesis or recycling mask a net loss. Because total glutathione does not distinguish reduced GSH from oxidized GSSG, our measurements cannot exclude changes in glutathione redox state. The more informative GSH/GSSG ratio is technically difficult to measure reliably because of rapid GSH oxidation during sample processing, and was not determined here. Thus, our data demonstrate that erastin-induced ferroptosis in flies can occur without detectable depletion of the total glutathione pool, while the contribution of reduced GSH and glutathione redox state remains to be established.

Despite this distinct redox organization, Gtpx proved essential for *Drosophila* viability, paralleling the essential requirement for GPX4 in mammals. Homozygous *Gtpx* loss was lethal, and dietary ferrostatin-1 did not rescue this phenotype. However, the absence of rescue does not establish whether *Gtpx*-deficient animals die through ferroptosis. Because *Drosophila* embryos do not feed, dietary ferrostatin-1 may not be available during a critical embryonic requirement for Gtpx; alternatively, Gtpx loss may compromise essential peroxide-control functions that are not prevented by ferrostatin-1. Thus, the genetic data support conservation of an essential function of GPX4/Gtpx in viability, while the mechanism underlying *Gtpx*-knockout lethality remains to be determined.

This raises the question of how erastin disrupts iron homeostasis without affecting glutathione. In male flies, erastin increased the pro-oxidative Fe^2+^/Fe^3+^ ratio and reduced ferritin-bound iron, consistent with increased availability of redox-active iron, even though total glutathione was unchanged. The best-characterized route by which erastin mobilizes iron in mammalian cells is NCOA4-mediated ferritinophagy, the autophagic degradation of ferritin that releases labile iron [65]. *Drosophila* lacks an NCOA4 ortholog (Fig. 1, Table 1), suggesting that the observed changes involve alternative mechanisms of ferritin turnover or iron mobilization that remain to be defined. That iron dysregulation occurs at all, despite the absence of the NCOA4 ferritinophagy pathway, indicates that the iron-centred arm of ferroptosis is driven in flies by mechanisms partly distinct from those defined in mammals, and makes *Drosophila* an informative model for dissecting this non-canonical, iron-dependent arm.

A separate question is whether flies possess a functional, erastin-sensitive system xc^−^. JhI-21 did not protect mammalian cells against erastin, alone or with the fly heavy chain CD98hc, suggesting either that we tested the wrong candidate or that no canonical system xc^−^ exists in *Drosophila*. We selected JhI-21 because it was the only light chain transcriptionally induced by erastin (Fig. 4E), reasoning that an erastin-responsive candidate was a plausible candidate for functional testing; sobremesa, however, was the fly sequence positioned closest to the vertebrate xCT clade in our tree, albeit with weak support (bootstrap 57), and was not tested here. Whether sobremesa or another candidate constitutes a bona fide fly xCT therefore remains open. We also cannot exclude that JhI-21 or CD98hc failed to reach the plasma membrane in mouse cells, since heavy-chain N-glycosylation is required for correct trafficking of the CD98–light-chain complex to the cell surface [66]; aberrant glycosylation of the fly heavy chain could produce the same negative result as a genuine absence of transport activity. Importantly, the absence of an identified canonical xCT ortholog does not preclude an erastin-sensitive ferroptotic phenotype, as erastin has additional cellular targets, including voltage-dependent anion channels [8,[67], [68], [69]] that are conserved in *Drosophila* [70].

We conclude that w^1118^ flies represent a valuable complement to existing mammalian models, offering several advantages: genetic tractability, rapid generation time, and the ability to study ferroptosis in a simplified system with distinct antioxidant and iron-regulatory architecture. Male flies are particularly informative for mechanistic studies because the ferrostatin-1-sensitive survival phenotype is accompanied by detectable changes in iron homeostasis and lipid composition. However, important limitations include the apparent absence of canonical system xc^–^, reliance on thioredoxin rather than glutathione systems, and sex-specific effects that may complicate interpretation. The model is particularly suited for studying iron-dependent mechanisms, ferroptosis-associated lipid remodeling, and genetic modifiers of ferroptosis susceptibility.

The establishment of *Drosophila* as a ferroptosis model expands the toolkit available to researchers and provides a genetically tractable system for mechanistic studies. While not replacing mammalian models, flies offer unique insights into the diversity of ferroptosis mechanisms and may reveal therapeutic targets not apparent in glutathione-dependent systems.

## CRediT authorship contribution statement

**Li Zhang:** Writing – review & editing, Visualization, Supervision, Investigation. **Felix Schmidt:** Writing – review & editing, Visualization, Supervision, Investigation. **Fatih Yasar:** Investigation. **Yasminn Akele:** Investigation. **Bernhard Michalke:** Investigation. **Konstantin Beckschäfer:** Investigation. **Christoph Thiele:** Investigation, Funding acquisition. **Eva-Maria Hanschmann:** Writing – review & editing. **Vivek Venkataramani:** Supervision, Investigation. **Axel Methner:** Writing – review & editing, Writing – original draft, Visualization, Project administration, Funding acquisition, Formal analysis, Conceptualization.

## Ethics declaration

This study was conducted in accordance with the ARRIVE (Animal Research: Reporting of In Vivo Experiments) guidelines. Ethics committee approval was not required. Fly work does not require ethical approval.

## Funding

This research was funded by the Deutsche Forschungsgemeinschaft to AM (461705066), BM/VV (461705388) and CT (461705208) through the Priority Program “Ferroptosis: from Molecular Basics to Clinical Applications” (SPP 2306). The sponsor was not involved in study design, in the collection, analysis and interpretation of data, in the writing of the report or the decision to submit the article for publication.

## Declaration of competing interest

The authors declare that they have no known competing financial interests or personal relationships that could have appeared to influence the work reported in this paper.

## Data Availability

Data will be made available on request.
