# Supplementary material for: The fruitfly Drosophila melanogaster as an in vivo model of ferroptosis
Source: Redox Biol. 2026 Sep 28;97:104418. doi: 10.1016/j.redox.2026.104418 (PMC13659589; doi:10.1016/j.redox.2026.104418)
Supplement: Supplementary file 1 [file mmc1.docx]

# Supplemental data

Supplemental figure 1: Lifespan of male and female Canton-S and Oregon-R *Drosophila melanogaster* strains. Each group consisted of 100 flies and was subjected to different concentrations of erastin (0 µM, 1 µM, 10 µM) mixed in 5% sucrose-based food.
